# Genetic rearrangements result in altered gene expression and novel fusion transcripts in Sézary syndrome

**DOI:** 10.18632/oncotarget.17383

**Published:** 2017-04-24

**Authors:** Grzegorz K. Przybylski, Claudia Gand, Floriane C. Braun, Piotr Grabarczyk, Andreas W. Kuss, Karolina Olek-Hrab, Armando N. Bastidas Torres, Maarten H. Vermeer, Willem H. Zoutman, Cornelis P. Tensen, Christian A. Schmidt

**Affiliations:** ^1^ Institute of Human Genetics, Polish Academy of Sciences, Poznan, Poland; ^2^ Clinic for Internal Medicine C, University Medicine Greifswald, Greifswald, Germany; ^3^ Department of Functional Genomics, University Medicine Greifswald, Greifswald, Germany; ^4^ Department of Dermatology, Karol Marcinkowski University of Medical Sciences, Poznan, Poland; ^5^ Department of Dermatology, Leiden University Medical Center, Leiden, The Netherlands

**Keywords:** Sézary syndrome, NGS, whole genome, RNASeq, rearrangement

## Abstract

Sézary syndrome (SS) is an aggressive, leukemic cutaneous T-cell lymphoma variant. Molecular pathogenesis of SS is still unclear despite many studies on genetic alterations, gene expression and epigenetic regulations. Through whole genome and transcriptome next generation sequencing nine Sézary syndrome patients were analyzed in terms of copy number variations and rearrangements affecting gene expression. Recurrent copy number variations were detected within 8q (*MYC, TOX*), 17p (*TP53, NCOR1*), 10q (*PTEN, FAS*), 2p (*DNMT3A*), 11q (*USP28*), 9p (*CAAP1*), but no recurrent rearrangements were identified. However, expression of five genes involved in rearrangements (*TMEM244, EHD1, MTMR2, RNF123* and *TOX)* was altered in all patients. Fifteen rearrangements detected in Sézary syndrome patients and SeAx resulted in an expression of new fusion transcripts, nine of them were in frame (*EHD1-CAPN12, TMEM66-BAIAP2, MBD4-PTPRC, PTPRC-CPN2, MYB-MBNL1, TFG-GPR128, MAP4K3-FIGLA, DCP1A-CCL27, MBNL1-KIAA2018*) and five resulted in ectopic expression of fragments of genes not expressed in normal T-cells (*BAIAP2, CPN2, GPR128, CAPN12, FIGLA)*. Our results not only underscored the genomic complexity of the Sézary cancer cell genome but also showed an unpreceded large variety of novel gene rearrangements resulting in fusions transcripts and ectopically expressed genes.

## INTRODUCTION

Sézary syndrome (SS) is an aggressive, leukemic cutaneous T-cell lymphoma variant [1]. It is characterized by the presence of atypical, malignant Sézary cells (CD4+CD45RO+) in blood, lymph nodes and skin, with phenotype of central memory T-cells (T_CM_) [2], severe erythroderma, pruritus and lymphadenopathy. SS is a very rare disease (incidence rate 0.1/100 000) and consists of 3% of cutaneous T cell lymphoma (CTCL) [1, 3], but with an increasing incidence rate [4]. Median age at presentation is 60 years and despite treatment the prognosis for patients is bad [5]. Data from the largest cohort of advanced Mycosis fungoides (MF) and SS patients (1,275) revealed that the medium overall survival (OS) is 63 months, and, depending on the four risk factors (stage IV, age > 60 years, large-cell transformation, and increased lactate dehydrogenase), the 5-year survival differs markedly from 68% for low risk group, through 44% for intermediate risk to 28% for high risk group [6].

Molecular pathogenesis of SS is still unclear despite many studies on genetic alterations, gene expression and epigenetic regulations. Both cytogenetic and molecular studies revealed chromosomal instability with recurrent “hotspots” of chromosomal abnormalities like 17p (loss of *TP53*) [7], 10q (loss of *PTEN* and *FAS*) [8, 9], 8q (gain of *MYC*) [10, 11], 9p (loss of *CDKN2A-CDKN2B*) [12], 19p (loss of *E2A*) [11] and 6q (loss of A20) [13]. Transcriptome analysis revealed genes being overexpressed in SS like *TOX* [14] or downregulated like *BIN1* [15] or *STAT4* [16], with a demonstrated influence on cell biology. Recently, Boonk et al. [17] demonstrated that loss of CD26 and/ or CD7 in combination with altered expression of *STAT4, TWIST1* and *DNM3* or *PLS3*, could distinguish SS patients from benign erythroderma cases with 100% specificity. Analysis on whole genomes of CTCL patients [18–21] identified mutations affecting genes involved in important signaling pathways (JAK-STAT, T-cell receptor (TCR) signaling), cell cycle control and epigenetic regulation (chromatin modifying genes, histone and DNA methyltranferases).

The purpose of this study was to combine the whole genome and transcriptome NGS technology, in order to look globally at genetic alterations and their effect on the expression of the affected genes in SS patients. Our results not only underscore the genomic complexity of the Sézary cancer cell genome and make evident that key signaling pathways in SS are abrogated but also show an unpreceded large variety of novel gene rearrangements resulting in fusions transcripts and ectopically expressed genes.

## RESULTS AND DISCUSSION

### Copy number variations (CNVs) within regions of oncogenes and suppressor genes and new “candidate” genes

**Figure 1:**
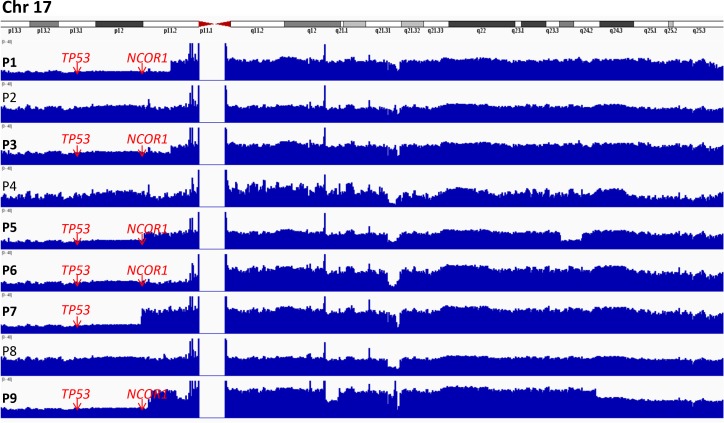
Integrative genomics viewer (IGV) visualization of alignments and coverage of the Illumina reads from the whole genome sequence analysis of 9 SS patients for chromosome 17 Whole genome analysis revealed deletions in the 17p region, involving *TP53* (P1, P3, P5-7, P9) and *NCOR1* (P1, P3, P5-6, P9). In patient P5 the break was located within *NCOR1* sparing the first exon.

**Figure 2:**
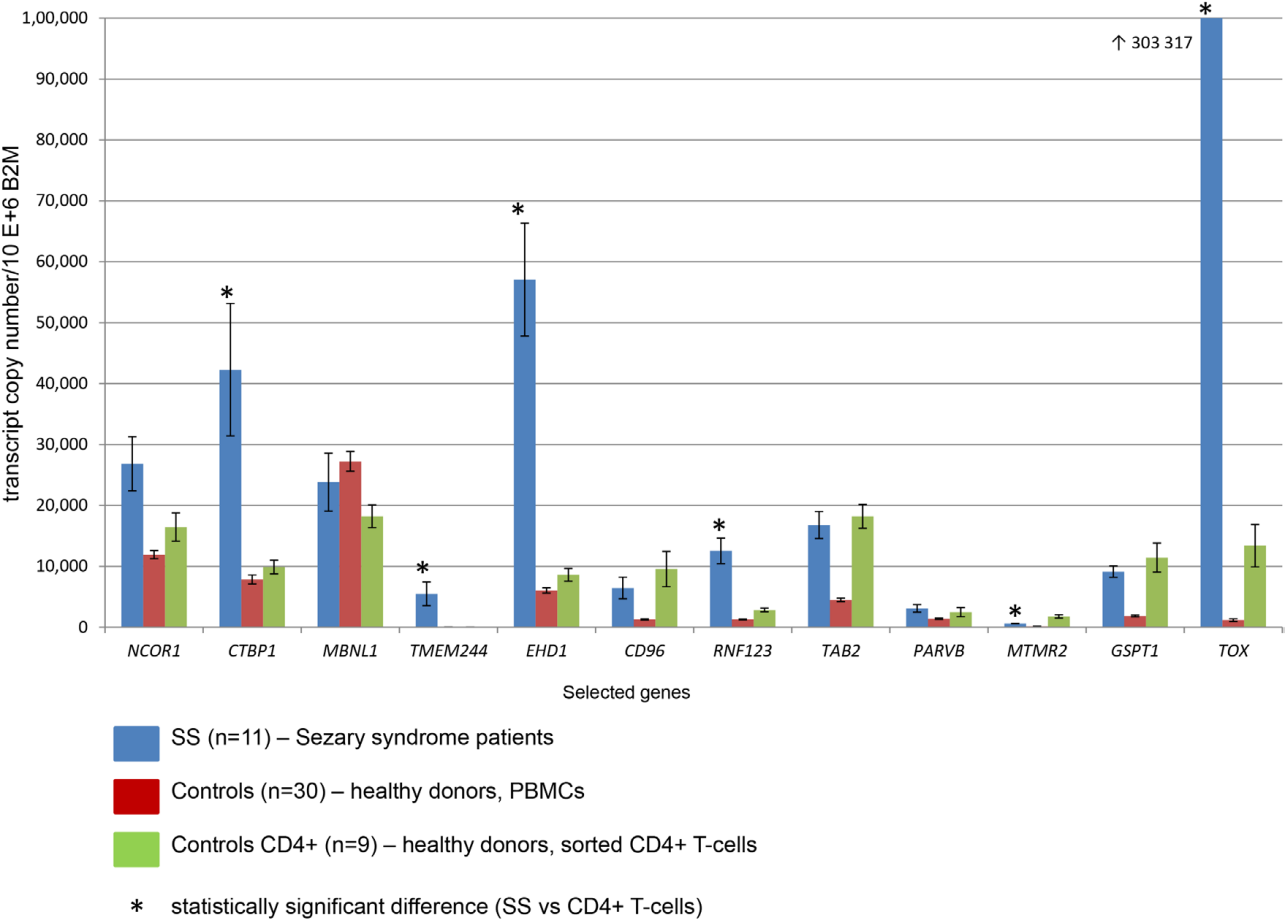
Expression of selected 12 genes measured by real-time quantitative PCR with specific TaqMan probes Standard errors are reported with bars.

Whole genomes of tumor cells of nine Sézary syndrome patients were examined by NGS in combination with inspection of these data using Integrative Genomics Viewer (IGV, Broad Institute). This analysis revealed many large deletions and amplifications (Supplementary Table 1). Recurrent CNVs occurred in regions of already known and described oncogenes, like *MYC* (8q amplifications in 4/9 patients) and suppressor genes like *TP53* (17p deletions in 6/9 patients) (Figure 1) and *PTEN* (10q deletions in 3 patients). *MYC* amplifications resulted in altered gene expression, especially in patient 5 with two extra copies of this gene (Reads per Kilobase per Million mapped reads - RPKM: C_n=10_=59.54; P5=224.05). *MYC* amplifications are common in SS [10, 11], but also in many other cancer types. Another gene, *TOX*, in the 8q region was shown to be highly expressed in SS and involved in abnormal cell proliferation [14]. Furthermore, in contrary to MYC, TOX was stated as a marker in differential diagnosis between SS and erythrodermic dermatitis [22]. Due to this reports, *TOX* expression was checked in our nine SS patients (RPKM: C_n=10_=5.1; SS_n=9_=63.0) and its overexpression was confirmed (Table 1). For three patients: P5 (RPKM=102), P8 (RPKM=155) and P9 (RPKM=88) this overexpression was the most significant, probably due to the presence of amplification in the 8q region. Strong upregulation of *TOX* in SS was confirmed by RQ-PCR on an additional cohort of SS patients. TOX showed the highest expression among the genes analyzed in SS, and was 21.88 times upregulated (p<0.0001) as compared to CD4+ controls (Figure 2).

**Table 1:** Expression of genes involved in rearrangements in SS and control samples (C)

Gene	Expression level (RPKM value)												RQ-PCR SS (11) vs CD4+ (9)
	C n=10	SS n=9	P1	P2	P3	P4	P5	P6	P7	P8	P9	SeAx	Fold Change;P value
*NCOR1* *(17p)*	38.0	29.6	**27.8** **Del**	52.8	**33.4** **del**	36.3	**24.6t**	**22.3** del	46.1	16.4	**6.8** **del**	**29.5** **del**	1.55; p=0.0937
	(p=0.1041)												
*CTBP1* *(4p)*	65.0	86.8	78.9	76	67.1	**61.2** **del**	75.4	139.5	**56.9** **t**	151.6	74.9	77.4	**3.35;** p=0.0004
	(p=0.0702)												
CD96 (3q)	**61.5**	**31.6** **↓**	9.0	18.4	15.1	**81.8** **dup**	31.7	43.5	**61.7** **t**	12.1	10.5	0.6	0.68; p=0.3658
	(p=0.0041)												
*MBNL1* *(3q)*	**249.8**	**131** **↓**	114	178	117.1	**233** **dup**	12	**110.6** **t**	261.9	14.74	32.8	**69.7** **tr**	1.12; p=0.6528
	(p=0.0009)												
*TAB2* *(6q)*	**87.3**	**67.9**	**31.1** **del tr**	47.6	49.0	52.2	51.2	42.9	205.9	32.5	98.4	24.1	0.89; p=0.5489
	(p=0.3049)												
*TMEM* 244 *(6q)*	**0.2**	**8.5** **↑**	**6.7** **t**	3.1	12.3	11.6	13.2	3.1	10.7	1.2	14.6	10	**500.6;** p<0.0001
	(p=0.0001)												
*EHD1* *(11q)*	**30.3**	**86.4** **↑**	**68.5** **t**	74.7	54.9	66.4	67.0	39.0	44.1	188.3	174.4	29	**6.19;** p<0.0001
	(p=0.0053)												
*MTMR2* *(11q)*	**11.0**	**5.0** **↓**	4.8	5.9	4.6	**3.3** **t**	5.5	4.4	4.9	7.1	4.6	4.7	**0.37;** p=0.0002
	(p=0.0042)												
*GSPT1* *(16p)*	**34.5**	**19.9**↓	22.2	23.0	21.2	**14.9** **t**	28	26.7	25.8	8.4	9.0	22.3	0.93; p=0.7606
	(p=0.0001)												
*PARVB* *(22q)*	**8.8**	**21.0** **↑**	31.6	16.9	17.5	27.0	12.2	18.9	**13.6** **t**	40.7	11.1	22.4	1.14; p=0.6840
	(p=0.0020)												
*RNF123* *(3p)*	**7.1**	**13.8** **↑**	17.8	11.9	12.5	12.8	**7.5** **t**	13.8	11.0	17.7	19.4	9.4	**4.08;** p<0.0001
	(p=0.0001)												
*TOX* *(8q)*	**5.06**	**63.0** **↑**	42.8	7.0	33.3	45.1	**102** **dup**	75.0	19.1	**154.5** **dup**	**88.1** **dup**	23.5	**21.88;** p<0.0001
	(p=0.0011)												

*TP53* deletions and mutations were described to be frequent in SS, and as a result this gene was suggested to be a cancer driver gene in Sezary syndrome [7, 18–21]. In recent papers alterations in *TP53* were detected in 24-92.5% of patients [18–20]. In this study monoallelic deletions of *TP53* resulted in decreased expression of this gene in 5 patients (RPKM: C_n=10_=22.73; P1=11.88; P3=10.71; P6=10.19; P7=11.62; P9=10.69), but in P5 increased expression was detected (RPKM=46.49). Mutations that may influence the function of TP53 were identified in P1 (TGT→TTT; C238F) and P5 (splicing site at the 5′ end of intron 7; GT→TT).

The 10q region is recurrently deleted in SS and the status of two genes in this region: *PTEN, FAS* have already been investigated. *PTEN* was described to be downregulated in SS, mostly by deletions [8]. In this study in 3 patients heterozygous *PTEN* deletions were found, with no significant influence on its expression (RPKM: C_n=10_=20.37; P1=14.6; P5=17.43; P7=29.33). The second gene, *FAS*, was deleted in 5 SS patients, but its expression was decreased in all of them (RPKM: C_n=10_=49.5; SS_n=9_=11.5). This is in agreement with previous studies showing that in majority of SS patients FAS expression was lost [9], not only because of recurrent deletions in this region, but also due to hypermethylation of CpG islands in the *FAS* promoter. Furthermore, inactivating *FAS* mutations are frequent in SS (10%-42.5%) [18, 19], confirming the putative role of different FAS alterations in malignant transformation and its possible role as a cancer driver. The 10p region is also frequently deleted in SS. Recent whole genome studies showed not only deletions, but also loss of function mutations (45.2%-65%) in Zinc Finger E-Box Binding Homeobox 1 (*ZEB1*) encoding an important T cell transcription factor [18–20]. In our study we identified deletions of *ZEB1* in 5 patients, including one biallelic in P7, all of them having an impact on *ZEB1* expression (RPKM: C_n=10_=21.5; P1=11.7, P5=13.8; P6=14.2; P7=2.5; P9=7.7).

Interestingly, deletions in the 6q region, that were described to be quite common in SS (50% of patients) [13], were detected only in P1 in this study, and it didn't include the tumor necrosis factor, alpha-induced protein 3 *(TNFAIP3)* gene. However, on the contrary to this previous study including pretreated patients, only tumor cells from newly diagnosed patients were studied in this one, suggesting *TNFAIP3* deletions to be late events in the SS progression. Late occurrence of *TNFAIP3* deletions is further supported by their absence in newly diagnosed Mycosis fungoides patients (unpublished data).

Deletions within 2p region resulted in a loss of DNA (cytosine-5-)-methyltransferase 3 alpha gene (*DNMT3A)* in four patients, biallelic in patient P8 and monoallelic in patients P1, P5 and P9, with a significant influence of its expression (RPKM: C_n=10_=15.32; P1=7.9; P5=6.41; P8=0.1; P9=7.13). In other SS patients the expression of *DNMT3A*, for other reasons, was also lower compared to controls (RPKM: SS_n=9_=8.33). According to previous studies *DNMT3A* and other *DNMT* genes were usually overexpressed in cancer [23], leading to hypermethylation, gene silencing and possible malignant transformation. However, in aging cells and age-related diseases global hypomethylation of the genome is detected [24], suggesting the possible decreased activity of DNA methyltransferases. Alterations in genes like *DNMT3A, DNMT3B, TET1, TET2* have recently been described to be very frequent in SS [18–21] and our results support a potential role of epigenetic modifiers in SS pathogenesis.

New candidate genes were investigated in commonly deleted regions. Three patients (P4, P5, P7) had monoallelic deletions of ubiquitin specific peptidase 28 gene (*USP28)* (11q) (RPKM: C_n=10_=10.91; P4=3.56; P5=4.27; P7=2.46). Genomic studies revealed that both, deletions and overexpression of *USP28* could be detected in human cancer [25]. *USP28* is a modulator of tumor suppressor FBW7 [26], but in a dose-dependent manner [25]. Only the complete knockdown or upregulation of *USP28* was proven to promote oncogenic transformation, due to FBW7 destabilization and accumulation of FBW7 substrates, like MYC [25]. On the contrary, partial deletions of *USP28* stabilize FBW7 and drives FBW7 substrates degradation, promoting tumor suppression. Therefore, in our samples *USP28* deletion is not likely to be the mechanism driving the malignant transformation, but rather the self-defense mechanism in those Sézary cells with the highest levels of *MYC* expression (RPKM: C_n=10_=59.54; P4=136.46; P5=224.05; P7=144.81).

Two patients (P1, P5) had caspase activity and apoptosis inhibitor 1 gene (*CAAP1)* (9p) deleted, the second patient on both alleles (RPKM: C_n=10_=12.92; P1=6.36; P5=0.93). Knockdown of *CAAP1* was proven to induce apoptosis in a caspase-10 manner [27], which is not observed in SS samples with decreased (P1) or lost (P5) *CAAP1* expression. One should keep in mind, that most likely p53 is non-functional in those two samples (due to LOH, see above), and since caspase-10 is activated in a p53-induced manner [27], this pathway could be blocked.

### Genes involved in rearrangements have altered expression in SS patients

A variety of rearrangements (306; Supplementary Table 2) were detected in SS patients. Most of them were unique for each patient. We decided to focus on those changes that affect the same genes and their expression in more than one patient.

*NCOR1* (nuclear receptor corepressor 1) is situated in the 17p region, which is one of the most commonly deleted regions in SS [20]. *NCOR1* deletions were detected previously in SS patients (38/80) [20], in some cases they were associated with mutations leading to biallelic loss of function. In this study *NCOR1* was fully deleted in four patients (P1, P3, P6, P9) (Figure 1) and in one patient (P5) most of the gene was deleted, except for exon 1, which was translocated to chromosome 15 and fused head-to-head with *SV2B* gene [chr15:91,813k/chr17:16,099 k]. *CTBP1* (C-terminal binding protein 1) (4p) was fully deleted in P4, while in P7 it was involved in a complex rearrangement: translocation t(2;4), [chr4: 1,241k (*CTBP1*i1)/chr2: 9,994k (*TAF1B*i4); chr2: 231,508k/chr4: 1,240k (*CTBP1*i1)], and t(4;11) [chr4: 1,240k/chr11: 76,788k (*CAPN5*i1)]. Both *NCOR1* and *CTBP1* have similar function in cells, they encode proteins involved in histone deacetylases (HDAC) complexes [28], which regulate HDACs activity. Despite the genomic alterations they seem to have higher mRNA expression in SS, yet only overexpression of *CTBP1* was confirmed by PCR (Table 1; Figure 2). Destabilization of HDAC activity can lead to imbalance in gene expression, which is observed in human diseases [29], including SS [30]. Since HDAC inhibitors revolutionized the treatment of CTCL patients [31], the status of HDACs in the pathogenesis of SS should be studied in details, also in terms of HDACs' interactions with proteins, regulating their activity.

**Figure 3:**
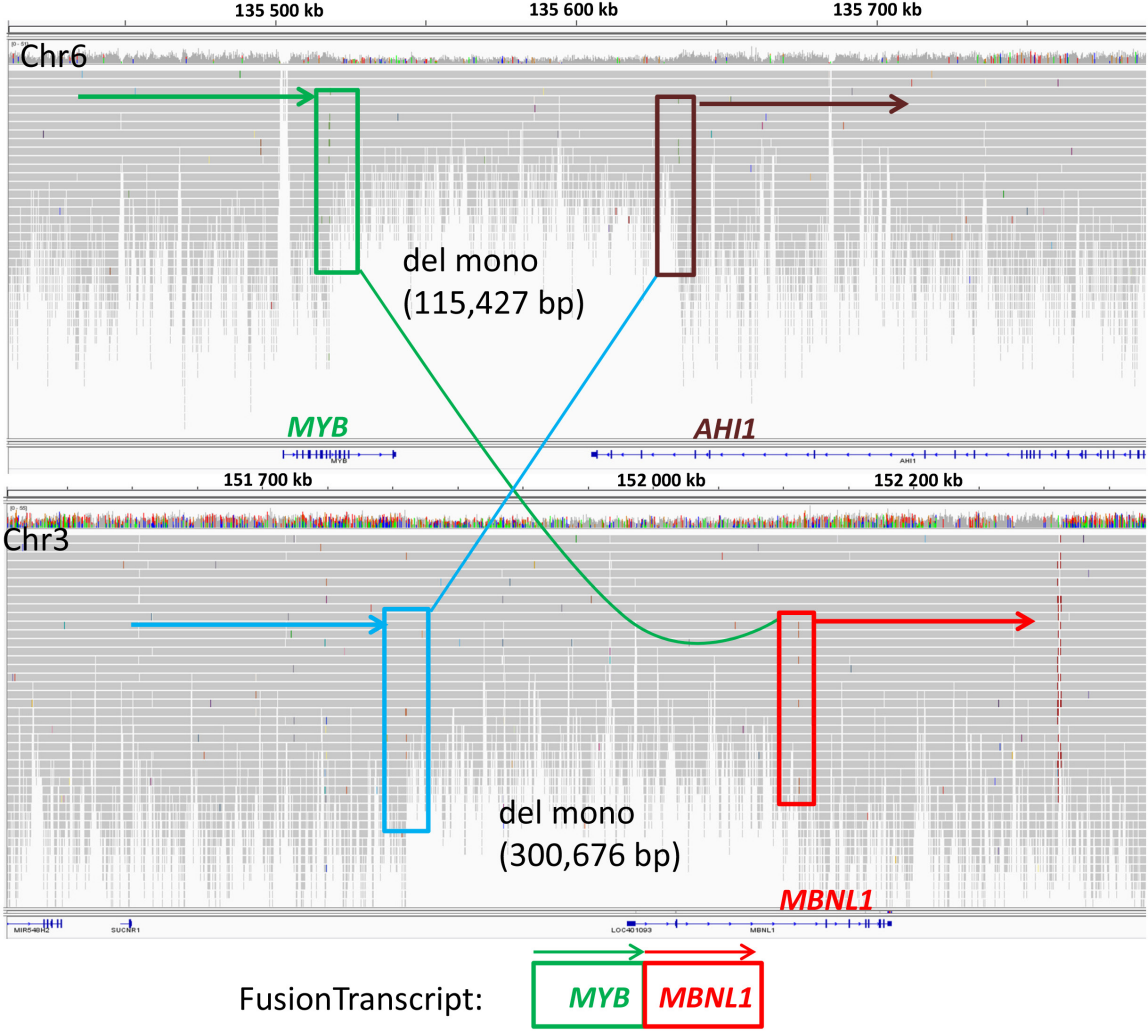
Integrative genomics viewer (IGV) visualization of alignments of the Illumina reads from the whole genome sequence analysis of SS patient (P6) for chromosome 6 (top) and 3 (bottom) Shown are two deletions, on chromosomes 6 and 3, associated with a reciprocal translocation t(3;6), in this SS patient. The 5′ part of *MYB* (green box) on chromosome 6 was fused to the 3′ part of *MBNL1* (red box) on chromosome 3, resulting in a in frame *MYB-MBNL1* fusion. An uncoding region of chromosome 3 (blue box) was rearranged to the 3′ part of *AHI1* (brown box). Regions of chromosome 3 (containing the 3′ part of *MYB* and the 5′ part of *MBNL1)* and chromosome 6 (containing the 5′ part of *MBNL1)* were deleted during the translocation.

*CD96* and *MBNL1* (muscleblind-like splicing regulator 1), both located in the amplified region in patient P4 (3q), were overexpressed in this sample (Table 2), on the contrary to other SS patients, where those genes were clearly downregulated. In another patient (P7) the 5′ end of the *CD96* gene was deleted, the coding region, except for the last exon 15 (rearranged within chr 3), was involved in complex translocation between chr 2 and chr 22 [chr2: 239,042k/chr3: 111,338k (*CD96*i9)-111,368k (*CD96*i14)/chr22: 44,503k (*PARVB*i4)]. *MBNL1* was involved in two gene fusions, in sample P6 and SeAx (see below). In P6 *MBNL1* fused with *MYB* as a result of reciprocal translocation (3;6) associated with deletions [chr6: 135,518,448 (*MYB*i9)/chr3: 152,111,859 (*MBNL1*i2); chr3:151,811,183/chr6:135,633,875 (*AHI1*i26)] (Figure 3). This is yet another example of *MYB* involvement in rearrangements and deletions in SS, noticed by our team [32]. In SeAx *MBNL1* was broken within intron 1, 5′ end was transpositioned in a close proximity to *KIAA2018* gene [chr3: 151,998,982 (*MBNL1*i1)/chr3: 113,432,006] and created the fusion transcript, while 3′ end fused tail-to-tail with the part of *MEDL12* gene [chr3: 152,000,045 (*MBNL1*i1)/chr3: 151,111,621 (*MEDL12*i36)]. *MBNL1* mediates an important role in pre-mRNA alternative splicing regulation and depletion of Mbnl protein was proven to misregulate this process [33]. Because both fusions with *MBNL1* gene downregulated its expression (Table 1), we decided to look up this gene in other SS patients. Although, RNAseq analysis clearly showed reduced expression in most SS patients compared to controls, it was not confirmed by RQ-PCR (Table 1; Figure 2).

**Table 2:** Fusion transcripts expressed in SS samples as a result of DNA rearrangements

Sample	Rearrangement	Breakpoints (DNA)	Transcript (RNA)
P1	t(11;19)	chr11: 64,628,862/chr19: 39,235,710	*EHD1*(e1,2) – *CAPN12*(e3→9)IN FRAME
P4	complext(11;16)	ch11:+95,529,671/ch11:+34,158,725-34,161,514/chr16:8 872 293	*CEP57*(e1)-*NAT10*(e22→e23+GTA)-*ABAT*(i14 chr16:8,872,293-8,872,087)
P4	complext(8;17)	chr8:+29,928,614/chr17:-79,602,847–~79,603,500*NPLOC4*/ch17:~45,068,130–45,062,900/ch17:~79,073,680*BAIAP2*	*TMEM66*(e1→e2)*-BAIAP2*(e7→e12?)
P5	t(9;19)	chr9: 137,760,563/chr19: 19,119,871	*C9orf9*(e1→e2)-*SUGP2*(e6→e12)NOT IN FRAME
P5	t(1;17)	ch1:+26,171,755/ch17:+65,161,538	*HELZ*(e1→e15)*-AUNIP*(e2→e5)NOT IN FRAME
P6	t(1;3)	chr3:-129,151,514/chr1:+198,647,866/	*MBD4*(e1→e6)*-PTPRC*(e3→e4→?)IN FRAME
P6	t(1;3)	chr1: 198,644,193/chr2:39,030,368-39,030,512/chr3: 194,062,664	*PTPRC*(e1→2)*-CPN2*(fr e2)
P6	t(3;6)	chr6:+135,518,448/chr3:+152,111,859	*MYB*(e1→e9)*-MBNL1*(e3→e10)IN FRAME
P6	transposchr3	chr3: 100,334,867/chr3:100,446,145	*TFG*(e1→e3)*-GPR128*(e2→e15)IN FRAME
P8	Transposchr2	chr2:68,543,463/chr2:39,049,173–39,050,512/chr2: 69,247,504	*ARMC6* (e1→8, fr e9 [→19,168,467])-*DHX57* (e17→fr i17)[→39,049,173]-*CNRIP*(fr i2) [68,543,463→68,540,755…]
P8	Transposchr2	chr2:71,014,668/chr2:39,563,447	*MAP4K3*(e1→6)*-FIGLA*(e3→5)IN FRAME
P8	t(3;15;9)	chr3: -53,370,567/chr15: -44,079,110-44,076,343*SERF2*/chr9: -34,664,314	Variant 1: *DCP1A*(e1,2,3)–*CCl27*(e2-3)IN FRAMEVariant 2: *DCP1A*(e1,2,3)–*CCl27*(e3)NOT IN FRAME
P9	del chr2	chr2:27,743,165-27,911,890	Variant 1: *AHDCI*(e1)-*WASF2*(e5→e9)NOT IN FRAMEVariant 2: *AHDC1*(e1)–(fr i1)[27,917,171–27,917,046 (125 bp)]–*WASF2*(e5→e9)NOT IN FRAME
SeAx	inv chr3	chr3:151,998,982/chr3:113,432,006	*MBNL1*(e1)-*KIAA2018*(e2→e7)IN FRAME
SeAx	t(1;16)	chr16: 11, 002,416/chr1: 29,065,400-29,065,342/chr1: 29,065,438	Variant 1: *CIITA*(e1→e10)–*YTHDF2*(e4,5)NOT IN FRAMEVariant 2: *CIITA*(e1→e11)–*YTHDF2*(e4,5)NOT IN FRAMEVariant 3: *CIITA*(e1→e11)-*YTHDF2*(e5)NOT IN FRAME

Several genes (*TMEM244, RNF123, PARVB, EHD1, MTMR2, GSPT1, TAB2)*, involved in a rearrangement in only one patient, were selected based on their expression pattern in all SS patients as determined by RNAseq (Table 1). To confirm the pattern, expression of those genes was studied using TaqMan probes on a different cohort of SS patients and controls (Table 1; Figure 2). Four of those genes: *TMEM244, EHD1, MTMR2, RNF123* had statistically significant difference in expression level compared to controls. *TMEM244* (transmembrane protein 244) was only expressed in SS, no expression was detected in healthy T-cells. Even in P1, where one copy of the *TMEM244* genewas involved in a complex rearrangement, it still had higher expression compared to controls (Table 2). The function of *TMEM244* is unknown, however expression of other genes from the same family (*TMEM176A, TMEM276B, TMEM16A)* have been already linked to cancer [34, 35].

### Novel fusion transcripts created as a result of rearrangements

Fifteen rearrangements detected in SS patients and SeAx resulted in an expression of new fusion transcripts (Table 2). Those transcripts, except for one (*TFG-GPR128*) [36], have never been detected before, either in SS, or any other malignancy. *TFG-GPR128* was detected in patients with atypical myeloproliferative neoplasms, but also in a small percent of healthy individuals [36]. This is in agreement with other studies showing that chimeric RNA can be expressed in healthy individuals [37], especially when two genes involved reside closer on the genome. Moreover, even oncogenic fusion transcripts, like *BCR-ABL*, can be detected in normal cells [38, 39]. Knowing that, 30 healthy donors were checked for all fusion-driving rearrangements detected in SS patients in this study, but no fusion transcripts were detected.

Nine of identified fusions (Table 2) were in frame, with no premature stop codons. Theoretically, they could create fully length new proteins. However, for unknown reasons, fusion partners were not always fully expressed, like in *EHD1-CAPN12*, where exon 9 was the last to be expressed in a fusion transcript. Interestingly, sometimes the breakpoint was not within the partner gene, yet the fusion was still created. In *DCP1A-CCL27*, the breakpoint on DNA was within intron 3 of *DCP1A*, followed by a part of *SERF2* gene (2, 767 bp), translocated approximately 1.6 kb upstream from *CCL27* gene. Similarly, in *MBNL1-KIAA2018*, the first exon of *MBNL1* gene was transpositioned 16, 500 kb upstream from *KIAA2018*, just to create the transcript with the 2nd exon. In this case the fusion occurred between parts of 5′UTR, so the partner gene *KIAA2018* was intact, but the regulatory elements of *MBNL1* led to its overexpression compared to other samples (RPKM: C_n=10_=12.1; SS_n=9_=13.7; SeAx=53.1). In *TMEM66-BAIAP2*, both genes on DNA where separated by a 6 kb insertion, including the small part of *NPLOC4* gene. The expression of *BAIAP2* gene ended probably with exon 12, as there was another rearrangement starting within intron 12: t(17;21) [79, 082k;44, 412k*PKNOX1*i1].

**Figure 4:**
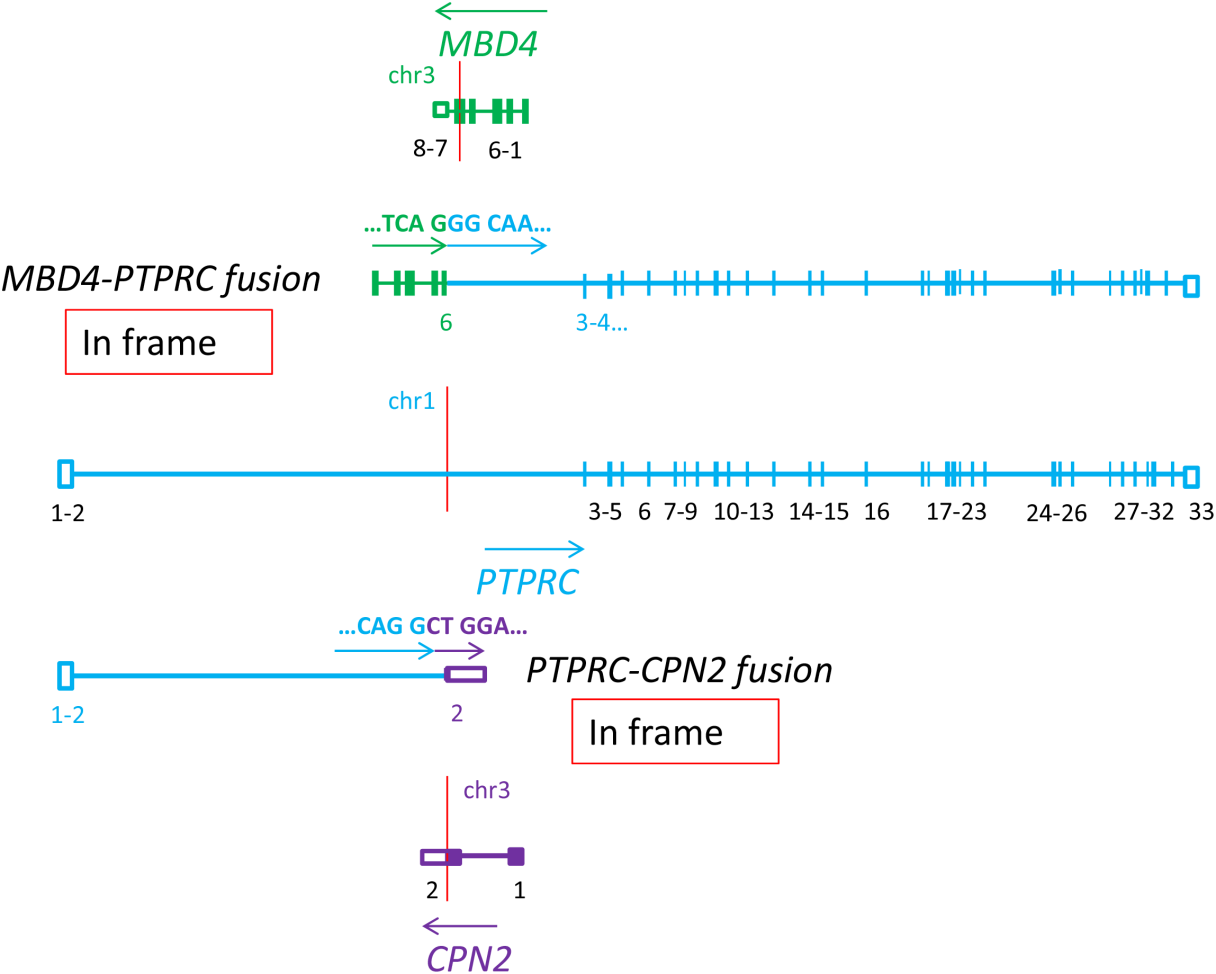
Schematic representation showing that the PTPRC gene, encoding the important T-cells antigen CD45, is involved in two in frame fusions: *MBD4-PTPRC* and *PTPRC-CPN2* (P6)

*PTPRC* was involved in two fusions (Figure 4). The breakpoint was within intron 2, first two exons were translocated to chr 3 and forced the expression of the fragment of the second exon of *CPN2* gene with 3′UTR. The second part of *PTPRC* gene was also translocated to chr 3, but under the influence of *MBD4* gene. *PTPRC* encodes the Protein Tyrosine Phosphatase, Receptor Type C, which is highly expressed in lymphocytes where it is also known as the CD45 antigen [40, 41]. By interaction with the LCK proto-oncogene, belonging to the Src family tyrosine kinases, PTPRC/CD45 is involved in TCR signaling pathways regulating T-cell growth, differentiation and function, and contributes to the malignant transformation [42, 43]. There are many variants of this gene expressed in T-cells, each with unique roles for signal transduction and apoptosis [44–46], therefore destabilization of this molecule could lead to lymphomagenesis.

Not in frame fusions (Table 2) had premature stop codons usually in the first exon after the breakpoint, probably leading to Nonsense-Mediated Decay (NMD) [47], which is a well-known mechanism preventing cells from accumulation of non-functional RNA and producing abnormal peptides.

**Figure 5:**
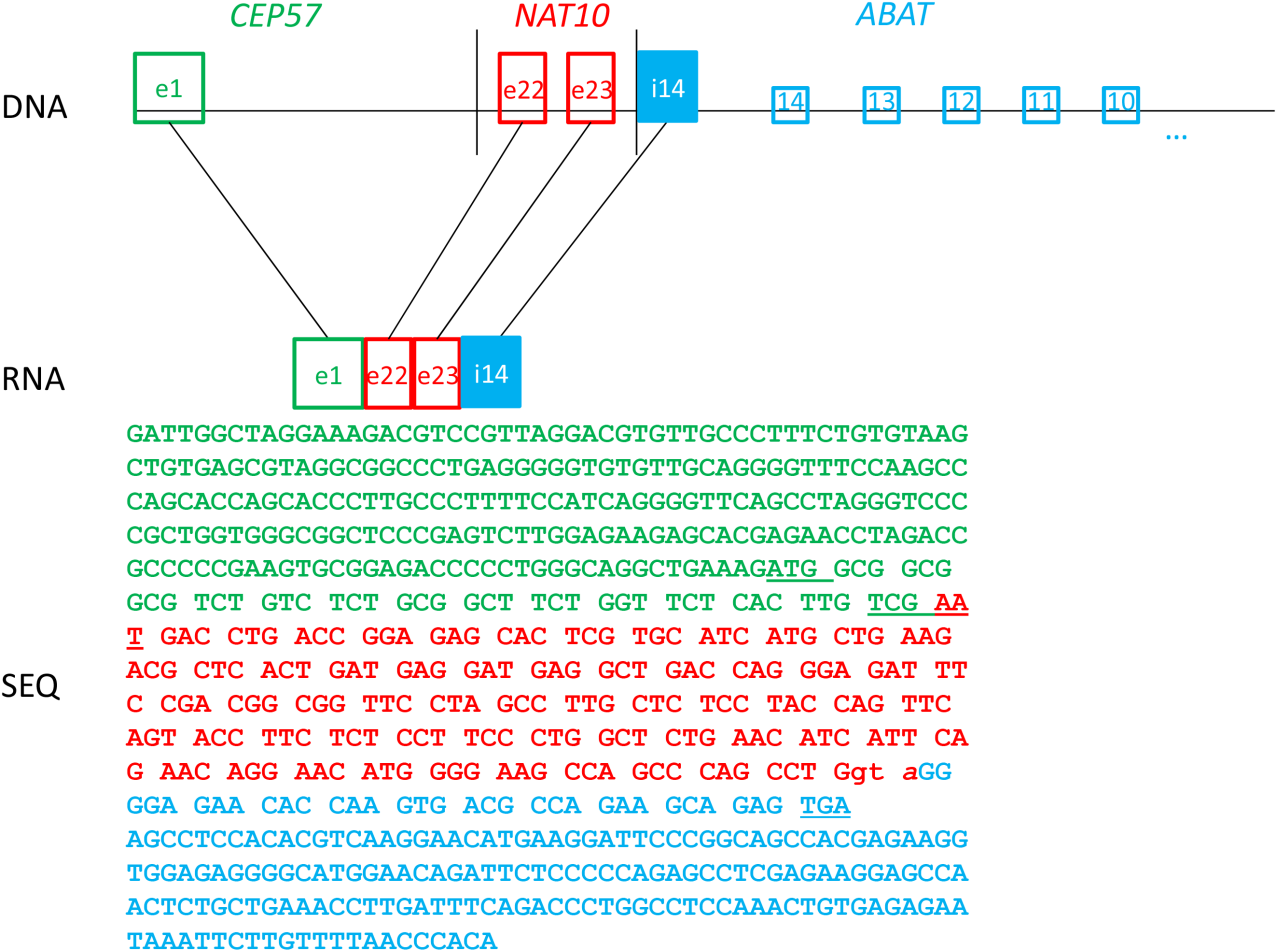
Schematic representation illustrating a complex rearrangement in patient P4 This rearrangement resulted in a fusion transcript between first exon of *CEP57*, two exons (22nd and 23d) of *NAT10* with trinucleotide insertion GTA, and a fragment of intron 14 of the *ABAT* gene.

Expression of chimeric RNA, with intronic elements have already been reported [37]. This whole transcriptome analysis revealed fusions with large intronic fragments: *CEP57-NAT10-ABAT* and *ARMC6-DHX57-CNRIP*. First exon of *CEP57* gene was fused in frame with exon 22 of *NAT10* gene (Table 2; Figure 5). Exon 23 was included in a fusion, but due to another breakpoint within the following intron, the expression was continued within intron 14 of *ABAT* gene (chr16). Twelve triplets encoding amino acids were present before the stop codon appeared, however, at this point it could not be determined whether it would be translated to a protein. In the second fusion, the transcription started with *ARMC6* gene, but interestingly it stopped in the middle of the 3′UTR, just to go on within genes *DHX57* and *CNRIP*. The implications of such expression remain unclear.

### Ectopic expression of gene fragments involved in gene fusions

Five gene fusions resulted in ectopic expression of fragments of genes usually not expressed in T-cells: *BAIAP2* (RPKM: P4=5; C_n=10_=0.74; SS_n=8_=1.54), *CPN2*, (RPKM: P6=24.63; C_n=10_=0.08; SS_n=8_=0.12), *GPR128*, (RPKM: P6=16.03; C_n=10_=0.06; SS_n=8_=0.07), *CAPN12*, (RPKM: P1=11.36; C_n=10_=0.60; SS_n=8_=0.76)*, FIGLA* (RPKM: P8=1.37; C_n=10_=0.07; SS_n=8_=0.10). Most of those genes, like *FIGLA*, have tissue specific expression. *FIGLA* is expressed in ovaries, as a central regulator of oocyte-specific genes that play roles in folliculogenesis, fertilization and early development [48]. In SS P8 *FIGLA* was ectopically expressed as a result of a fusion with *MAP4K3*, that have already been described as a fusion partner and a tumor suppressor in cancer [49, 50], and, in general, as an important player in a cell signal transduction.

### Conclusion

In conclusion, with this NGS analysis we showed that the variety of genetic events in Sézary cells is enormous, though common denominators could not be identified. It appears that malignant transformation is a result of alterations on many levels: genomic (mutations, CNVs, rearrangements), transcriptomic (elevated expression or downregulation, fusion transcripts, ectopic expression) and epigenomic (methylation, HDACs activity). Although, it is still unclear, which changes are the drivers that caused the disease, and which are the passengers that occurred during the disease progression, this study strongly confirms the role of known tumor suppressors (*TP53, FAS*), epigenetic modifiers (*NCOR1, DNMT3A*) and dysregulated signaling pathways (*PTPRC* in TCR signaling) in SS pathogenesis. Due to the huge variety of alterations we hypothesize that in case of SS different pathways lead to the same clinical presentation. SS is a disease of elderly, so different molecular changes could accumulate during lifetime in skin homing T_CM_, that persist long term in normal skin following resolution of an immune response [2, 51]. In the future, it would be worth to analyze SS patients individually, to classify them, based on their molecular profile, to choose the best treatment options.

## MATERIALS AND METHODS

### Clinical samples

Nine SS blood samples were included in the study: P1 (23) (F, age: 80), P2 (19) (M, age: 65), P3 (33) (M, age: 54) from the Department of Dermatology, University of Medical Sciences, Poznan, Poland and P4 (WZ1) (F, age: 74), P5 (WZ4) (M, age: 70), P6 (WZ8) (F, age:71), P7 (WZ9) (M, age: 59), P8 (3-0030) (M, age: 73), P9 (3-0044) (F, age: 64) from the Department of *Dermatology*, Leiden University Medical Center, Netherlands and the SS derived cell line (SeAx). Patient clinical information is given in Supplementary Table 3. Blood samples were collected once the diagnosis of SS was confirmed. The use of human material was approved by the Local Ethics Committees of both universities, and performed in accordance with the Declaration of Helsinki. Samples were used to extract DNA and RNA, three of them were Ficoll purified mononuclear cells (PBMCs) (P1-P3) while others were sorted CD4^+^ T-cells (P4-P9). PBMCs were enriched for CD4+ T cells by depletion of non-CD4+ T cells using the CD4+ T Cell Isolation Kit I (Miltenyi Biotec, Bergisch Gladbach, Germany), resulting in a cell purity >95% as claimed by the manufacturer [52]. The purity of the samples was confirmed by quantitative NGS analysis of the T cell receptor alpha/delta locus (Supplementary Figure 1). In samples P4-P9 no germline *TCRAD* sequence was visible, indicating almost 100% purity of the samples, while in samples P1-P3 an admixture of non-malignant cells, without *TCRAD* rearrangements, was present.

### Healthy individual control samples

For RNASeq 10 RNA samples from five healthy individuals (males: 24, 24, 28, 61 and 67 years old), isolated from sorted peripheral blood CD4+ or IL-2 activated CD4+ cells, were used as controls.

For RQ-PCR 9 RNA samples isolated from sorted peripheral blood CD4+ cells from healthy individuals (males: 21, 24 and 46 years old; females: 33, 33, 36, 49, 58 and 62 years old), and 30 RNA samples isolated from Ficoll purified mononuclear peripheral blood cells from healthy individuals (41-57 years old) were used as controls.

### Paired-end next-generation sequencing

Paired-end 15x coverage whole genome NGS was performed by BGI-Hong Kong on the HiSeq2000 Illumina platform. At least two non-identical inserts with discordant 5′ and 3′ reads localized in different regions of the genome were considered to carry a rearrangement.

### RNAseq

Paired–end RNASeq analysis was performed using two different NGS platforms: for 7 SS patients (P1-P7), SeAx and 10 control samples SOLID5500 platform was used (Life Technologies; mRNA fragments: 100-150 bp; reads: 75 bp/35 bp), while two other SS patients (P8, P9) were analyzed on HiSeq2000 Illumina platform (mRNA fragments: 150 bp reads: 100 bp).

### NGS data analysis

Sequence analysis was based on GRCh37/hg19. Sequences, as bam files and bam.tdf files, were analyzed using Integrative Genomics Viewer 2.3 (IGV 2.3, Broad Institute). RPKM values were used to measure mRNA abundance. Lifescope software was used to find fusion transcripts: Paired-end reads that mapped to different genes were considered as evidence of fusion transcripts.

### PCR, RT-PCR

Selected rearrangements and fusion transcripts were confirmed on DNA and cDNA using standard PCR and RT-PCR. Products were Sanger sequenced (Institute of Biochemistry and Biophysics PAS, Warsaw, Poland) to confirm the breakpoint. 30 DNA samples from healthy donors were used as controls.

### Real-time quantitative PCR (RQ-PCR)

RQ-PCR was performed using the CFX96 Real-Time System (BioRad, Hercules, CA). The following genes were analyzed by commercially obtained TaqMan Gene Expression Assays (Applied Biosystems, Foster City, CA): *NCOR1* (Hs01094540_m1), *CTBP1* (Hs00972284_m1), *TAB2* (Hs00248373_m1), *CD96* (Hs00175524_m1), *EHD1* (Hs00199030_m1), *GSPT1* (Hs01093019_m1), *MTMR2* (Hs01547438_m1), *PARVB* (Hs00203381_m1), *RNF123* (Hs00222902_m1), *TMEM244* (Hs02340633_m1), *MBNL1* (Hs02569862_s1), *USP28* (Hs00363603_m1). Beta-2 microglobuline (*B2M)* (Hs00984230_m1), not affected by alterations and suitable for CD4+ lymphocytes, [53] was used as a reference gene for sample normalization. cDNA samples from 11 SS patients, 30 Ficoll purified peripheral blood mononuclear cell samples (PMBCs) from healthy donors and 9 sorted CD4^+^ T-cell samples from healthy donors were analyzed. All samples were assayed in duplicate. Statistical analysis of the differences in the gene expression was performed using two-tailed Student's t-test for unpaired data (GraphPad Software) and the 2^−ΔΔCT^ Method [54].

## SUPPLEMENTARY MATERIALS FIGURES AND TABLES


