# Supplementary material for: Genetic rearrangements result in altered gene expression and novel fusion transcripts in Sézary syndrome
Source: Oncotarget. 2017 Apr 24;8(24):39627–39. doi: 10.18632/oncotarget.17383 (PMC5503638; doi:10.18632/oncotarget.17383)
Supplement: Supplementary file 1 [file oncotarget-08-39627-s001.pdf]

Genetic rearrangements result in altered gene expression and novel fusion transcripts in Sézary syndrome

SUPPLEMENTARY MATERIALS

Quantitative Integrative genomics viewer (IGV) visualization of the T cell receptor alpha/delta locus coverage

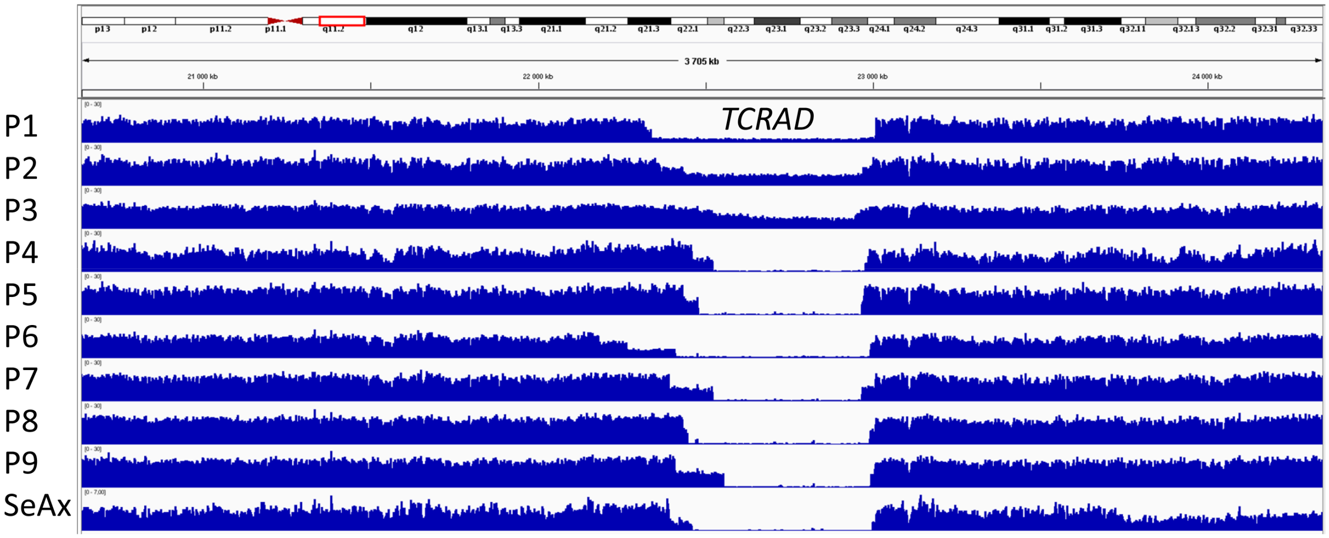

Supplementary Figure 1: *TCRAD* locus read coverage, indicating the purity of the of the Sézary syndrome samples. In samples P4-P9, similarly to the SeAx cell line, no germline *TCRAD* sequence is visible, indicating almost 100% purity of the samples. In samples P1-P3 an admixture of non-malignant cells, without *TCRAD* rearrangements, is present.

Supplementary Table 1: Copy number variations (CNVs) in nine SS patients

See Supplementary File 1

Supplementary Table 2: The list of all rearrangements detected in SS patients and SeAx cell line

See Supplementary File 2

Supplementary Table 3: Patient clinical information

Patient	Sex	Age	WBC count/nl	Lymphocytes count/nl [%]	atypical Sézary cells [%]	CD4/ CD8 ratio	CD4/ CD3 [%]	Therapy prior to sample collection	Follow up (months)
**P1**	F	80	24.8	18.3<br>73.6%	n/a	15.9	95%	Fexofenadine,<br>Levocetirizine,<br>Clemastine,<br>Levothyroxine	D+28 m
**P2**	M	65	9,5	1.5<br>15.3%	n/a	7.2	83%	Acitretin<br>Methylprednisolone<br>Omeprazole	A+48 m
**P3**	M	54	12.5	6.1<br>49%	n/a	n/a	n/a	PUVA<br>Methylprednisolone<br>Methotrexate	A+56 m
**P4**	F	74	25	10.5<br>42%	30%	206	100%	Local steroids	D+ 16 m
**P5**	M	70	28	12.6<br>45%	n/a	58	98%	Local steroids	D+ 24 m
**P6**	F	71	10	2.9<br>29%	26%	89	102%	Prednisone<br>30 mg daily	D+ 35 m
**P7**	M	59	11.3	5.8<br>51%	n/a	60	98%	Prednisone<br>30 mg daily	D+ 86 m
**P8**	M	73	31.9	8.6<br>27%	27%	121	99%	Bath PUVA	A+ 40 m
**P9**	F	64	45.4	15.9<br>35%	31%	183	99%	None	A+ 36 m

WBC: white blood cells
n/a: not assessed
PUVA: Psoralen Ultra-Violet A
A: alive
D: dead
